# Association of body-mass index with physiological brain pulsations across adulthood – a fast fMRI study

**DOI:** 10.1038/s41366-024-01515-5

**Published:** 2024-03-29

**Authors:** Lauri Raitamaa, Joona Kautto, Johanna Tuunanen, Heta Helakari, Niko Huotari, Matti Järvelä, Vesa Korhonen, Vesa Kiviniemi

**Affiliations:** 1 Oulu Functional NeuroImaging (OFNI), Diagnostic Imaging, Medical Research Center (MRC), Finland Oulu University Hospital, 90029 Oulu, Finland; 2 Research Unit of Health Sciences and Technology (HST), Faculty of Medicine, University of Oulu, 90220 Oulu, Finland; 3 Oulu Center for Cell-Matrix Research, Faculty of Biochemistry and Molecular Medicine, Biocenter Oulu, University of Oulu, Oulu, Finland

**Keywords:** Obesity, Translational research

## Abstract

**Background/objective:**

Obesity is a risk factor for several brain-related health issues, and high body-mass index (BMI) is associated with an increased risk for several neurological conditions, including cognitive decline and dementia. Cardiovascular, respiratory, and vasomotor brain pulsations have each been shown to drive intracranial cerebrovascular fluid (CSF) flow, which is linked to the brain metabolite efflux that sustains homeostasis. While these three physiological pulsations are demonstrably altered in numerous brain diseases, there is no previous investigation of the association between physiological brain pulsations and BMI.

**Subjects/methods:**

We measured the amplitudes of the physiological brain pulsations using amplitude of low frequency fluctation (ALFF) based method with resting-state functional magnetic resonance imaging via high temporal resolution whole-brain magnetic resonance encephalography (MREG) in 115 healthy subjects. We next undertook multiple linear regression to model the BMI effect voxel-wise whole-brain on very low frequency (VLF), respiration, cardiovascular, and respiratory induced modulation of cardiovascular pulsation amplitudes with age, pulse pressure, and gender as nuisance variables.

**Results:**

In our study population, BMI was positively associated with the amplitudes of vasomotor, respiratory, and respiratory induced modulations of cardiovascular pulsations (*p* < 0.05), while negatively associated with the amplitudes of cardiovascular pulsations (*p* < 0.05).

**Conclusions:**

The findings suggest that BMI is a significant factor in alterations of cardiovascular pulsation of neurofluids. As physiological pulsations are the drivers of CSF flow and subsequent metabolite clearance, these results emphasize the need for further research into the mechanisms through which obesity affects brain clearance.

## Introduction

Obesity has become a prevalent global public health concern, with a significant portion of the adult population now being overweight or obese [1]. The obesity epidemic is associated with decreased quality of life, burgeoning costs of health care, and rising mortality [2]. The relationship between height and body weight, typically quantified by body mass index (BMI), is a well-established index of obesity, where BMI > 25 shows a clear association with risk for chronic health and life-limiting conditions including cardiovascular disease, cerebrovascular disease, and respiratory complications [3].

High BMI is associated with declining cognitive ability in middle aged, and increases the risk for developing Alzheimer’s disease (AD) later in life [4]. Obesity can induce chronic brain inflammation, which may facilitate the formation of amyloid plaques and neurofibrillary tangles, which are the characteristic features of AD neuropathology [5, 6]. Additionally, obesity deleteriously affects the cerebral vasculature by promoting atherosclerotic cholesterol buildup in the vessel walls, leading not just to increased risk for cerebrovascular accident, but also to reduced arterial wall pulsatility, restricted blood flow, and microvessel damage, all of which are intricately connected to neurodegeneration processes [7]. While there is a strong association between high BMI and high blood pressure, they remain independent risk factors for dementia and cardiovascular disease [4, 8].

Besides cardiovascular changes, obesity also causes various respiratory problems [9], notably by imposing a higher workload on breathing due to increased airway resistance and reduced respiratory compliance [10]. Elevated intra-abdominal pressure due to visceral fat deposition reduces the respiratory system’s residual capacity, which in turn increases the force required for breathing [11]. These respiratory efforts can impact reciprocal venous return from the brain and cerebrospinal fluid (CSF) in/outflow dynamics from spinal canal [12, 13]. Imaging studies have revealed enlarged perivascular CSF spaces in brain of individuals with obesity, particularly those with sleep apnea or idiopathic intracranial hypertension (IIHT) [14, 15]; the increased CSF/venous blood flow pressure changes may result in impaired perivenous CSF flow.

In humans, respiratory and vasomotor waves are the primary drivers of CSF convection [16], increasing in intensity during sleep to facilitate glymphatic clearance [17]. Regarding the connection between obesity and brain diseases, MREG scans have identified abnormal brain pulsations and variations in AD, demonstrating significant clinical relevance [18, 19]. Moreover, early alterations in physiological brain pulsations show potential as predictive biomarkers for disease progression, notably in primary CNS lymphoma, where early pre-treatment MREG scans have been capable of predicting treatment outcomes [20]. In narcolepsy, cardiac and respiratory pulse variance decreases, while VLF increases compared to controls [21]. For epileptic patients, there’s an increase in respiratory pulsation power and synchrony, but a notable decrease in pulse propagation speed during exhalation, both in medicated focal epiletic and drug-naive seizure patients, relative to healthy individuals [22, 23]

The aim of this study was to investigate the relationship between BMI and physiological brain pulsations, using high temporal resolution whole-brain magnetic resonance encephalography (MREG), which allows simultaneous recording of the multiple drivers of brain fluid dynamics and avoiding the risk of misinterpreting results through inappropriate aliasing of higher frequency signal sources at lower frequency bands. MREG captures slow (circa 1 Hz) vasomotor waves and respiration pulsations from the venous blood oxygenation level dependent (BOLD) signal, and detects higher frequency CSF space and blood flow dynamics including arterial impulses, this without temporal aliasing [16]. Given the multiple physiological processes that are compromised by obesity, we hypothesized that elevated BMI would associate with impairment of the physiological brain pulsations driving human brain hydrodynamics. To investigate this hypothesis, we analyzed the amplitudes of each physiological pulsations in healthy subjects across a wide adult age range, and utilized multiple linear regression to explore their relationship with BMI. Our findings revealed significant associations between BMI and distinct physiological brain pulsations within specific brain regions.

## Materials and methods

### Participants

One hundred and twenty-four (124) healthy subjects participated in the study (Table 1). The subjects enrolled in the study reported themselves as healthy, with no history of neurological diseases or brain surgery. They were non-smokers, confirmed that they were not taking any continuous medication, and stated that they were not pregnant. Subjects were instructed to abstain from alcohol for at least 12 h prior to the scanning. Additionally, subjects were confirmed to have no MRI scanning contraindications based on Oulu University Hospital patient records. Subjects were instructed to lie still, with their eyes kept open and gaze fixated on a cross on a video screen, without thinking of anything in particular (eyes open, resting state). Ear plugs were provided to reduce perception of scanner noise. Cushions were placed beside the ears to restrict head movement and to further reduce scanner noise. During image preprocessing, three subjects were excluded because of partial data corruption, and six subjects were excluded because of excessive head movement, leaving 115 subjects to the study. Blood pressure was measured in a supine position just before the scan. Written informed consent was obtained from all participants according to the Declaration of Helsinki. The research was approved by the Regional Ethics Committee of the Northern Ostrobothnia Hospital District.

**Table 1:** Demographic information includes age, gender, height, weight, body-mass index (BMI), and blood pressure (systolic, diastolic, and pulse pressure).

Parameter	Range	Group mean ± SD
Age (years)	19–74	45.3 ± 16.4
Gender (Female/male ratio)		76/39
Height (cm)	152–200	169.7 ± 9.2
Weight (kg)	49–140	74.9 ± 15.1
Body-mass index (kg/m^2^)	17.4–38.5	25.9 ± 4.1
Systolic blood pressure (mmHg)	104–187	141.8 ± 20.1
Diastolic blood pressure (mmHg)	51–108	79.2 ± 11.6
Pulse pressure (mmHg)	39–97	62.6 ± 12.9

Descriptive statistics of range, mean, and standard deviation (SD) are provided to offer a comprehensive overview of the participant sample.

### Data acquisition and preprocessing

Subjects were scanned using a Siemens MAGNETOM 3 T SKYRA scanner with a 32-channel head coil. Additional cardiorespiratory data were collected using an MRI-compatible multimodal neuroimaging Hepta-Scan concept [24]. MREG is a 3D single shot stack of spirals (SOS) sequence that under-samples k-space to reach a sampling rate of 10 Hz, thus allowing critical imaging of physiological pulsations [25]. The SOS gathers k-space in 80 ms bins with spiral in/out repeating continuously in every other turn in the positive z-direction, thus minimizing the air-sinus off-resonance artifact [25]. The point spread function of the SOS-sequence is 3 mm, with lesser off-resonance effects compared to other k-space undersampling strategies such as concentric shells and spokes [25, 26]. MREG scanning parameters were TR = 100 ms, TE = 36 ms, flip angle = 5°, 3D matrix = 643, FOV = 192 mm with voxel size of 3 x 3 x 3 mm^3^, and for anatomical 3D MPRAGE, the parameters were TR = 1900 ms, TE = 2.49 ms, TI = 900 ms, flip angle = 9°, FOV = 240 mm, 0.9 mm cubic voxel. Cardiorespiratory frequencies were verified with an anesthesia monitor (GE Date-Ohmeda Aestive 5) and from scanner physiological data recordings using fingertip PPG/SpO_2_ and respiratory belt monitors.

MREG data were reconstructed using L2-Tikhonov regularization with lambda 0.1, where the latter regularization parameter was determined by the L-curve method with a MATLAB recon-tool from the sequence developers [27]. Spin saturation effects were minimized by deleting the 14 s epoque from the beginning of each scan.

For data analysis and processing, AFNI (Analysis of Functional NeuroImages, version 18.0.05) and FSL (Functional Magnetic Resonance Imaging of the Brain’s software library, version 5.0.9) were utilized [28, 29]. The AFNI *3dDespike* function was used to remove motion produced spikes from the remaining data. Next, we preprocessed the data with the standard FSL pipeline, using high-pass filtration with a cut-off frequency of 0.008 Hz (125 s). Motion correction was performed using FSL MCFLIRT, and FSL BET was used for brain extraction. The anatomical 3D MPRAGE images were used for registeration of MREG data into MNI152 standard space using FSL FLIRT. To minimize unwanted movement-related effect, subjects with a mean absolute movement >0.3 mm or having any timepoints with average movement >1.5 mm were excluded from the study. Subsequently nuisance head motion was removed in the MREG data using multiple linear regression was performed using FSL *fgl_glm* function to remove the six head realignment parameters (3 translation and 3 rotation) produced by MCFLIRT.

### Analysis of amplitude of fluctuation

**Fig. 1:**
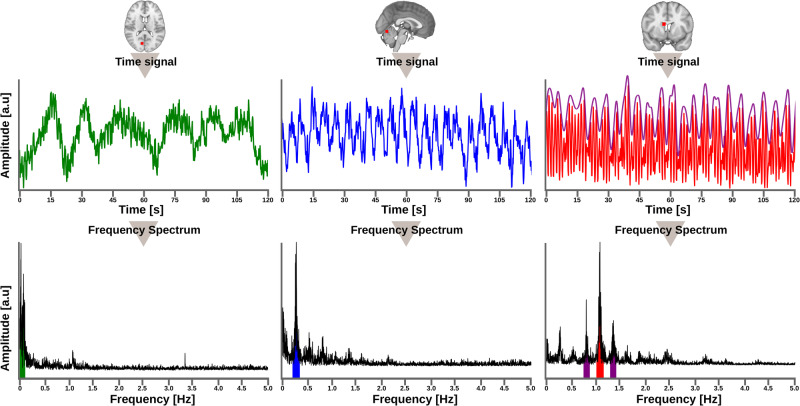
Three distinct raw MREG signals are featured, each featuring the characteristics of a studied physiological signal: very low frequency (VLF) in green, respiration in blue, and cardiovascular in red. These signals were extracted from three separate 3 mm diameter spherical regions of interest (ROIs), as indicated by the grey-scale brains with highlighted red dots in the upper section of the image. Additionally, cardiorespiratory modulation (CREM) is shown with cardiovascular signal as a purple envelope over the cardiac pulsations on right. The corresponding frequency spectrum is depicted below each time signal. Within these spectra, shaded colored regions indicate the chosen amplitude fluctuation (AF) bands for each studied frequency component: AF_VLF_ in green, AF_resp_ in blue, AF_card_ in red, and AF_CREM_ in purple.

We used a modified ALFF method to study amplitude of fluctuation (AF) of very low frequency (AF_VLF_), respiratory (AF_resp_), and cardiovascular (AF_card_) pulsations [16]. The frequency band for AF_VLF_ was 0.01–0.1 Hz, while the bands for AF_resp_ and AF_card_ were 0.1 Hz wide, centered around the individual bands (i.e., center of the band ± 0.05 Hz indicated in colors; Fig. 1). To define the respiratory-induced modulation of cardiovascular pulsation, we quantified it as the amplitude of cardiorespiratory modulation, employing the same methodology as used in our previous study (Fig. 1, purpe bands) [16]. For every subject, the time courses of each voxel from whole brain temporal MREG data were transformed using AFNI 3dPeriodogram to the frequency domain via a fast Fourier transformation, which yielded the voxel-wise power spectrum. The square root of the power spectral density was calculated, and amplitudes calculated over the frequency bands of interest were summed to obtain a corresponding AF map. The AF value in any given voxel represents the total voxel-wise amplitude of a chosen frequency band [30].

### Statistical analysis

Our primary objective was to assess the relationship between BMI and physiological brain pulsations. Voxel-wise comparisons between different AF maps were performed by a two-sample *t*-test using a non-parametric threshold-free permutation test (5000 permutations) implemented in randomize from FSL [31]. A multiple linear regression model was used to assess the association between BMI and AF metrics, in which each subject’s age, sex, and pulse pressure were included as nuisance variables. As pulse pressure is an independent predictor for risk of cardiovascular disease, and to minimize the effect of linearity, we opted to use pulse pressure instead of systolic and diastolic pressure as a nuisance variable. Using pulse pressure the variance inflation factor of the model was only 1.36, thus well below the cut-off of 5 that is generally considered an indication of moderate multicollinearity [32]. In the voxel-based statistical tests we used a whole brain mask including white matter, grey matter, and CSF. The tests were corrected for the family-wise error rate (FWER) at a significance level of *p* < 0.05. All data were reported as mean ± standard deviation (SD) unless otherwise stated.

## Results

### Very low frequency < 0.1 Hz vasomotor brain pulsation

**Fig. 2:**
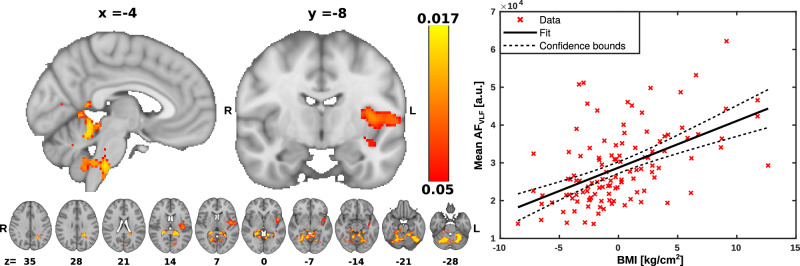
Correlation between body mass index (BMI) and very low frequency (VLF) amplitude of fluctuation (AF_VLF_) in brain regions. **Left** Statistical map of the voxelwise correlation between AF_VLF_ with BMI. AF_VLF_ was positively associated with BMI in brain regions indicated on the red-yellow color scale. Significance was assessed using family-wise error rate (FWER) correction at *p* < 0.05. R right, L left, *x* displayed sagittal plane, *y* displayed coronal plane, *z* displayed axial plane. **Right** Correlation plot illustrating the relationship between mean AF_VLF_ averaged across significant (*p* < 0.05) brain areas as a function of BMI (R^2^ = 0.268, *p* < 0.001).

To investigate the association between VLF brain pulsations and BMI, we used the multiple linear regression model to assess voxel wise the association of BMI to AF_VLF_. For a further exploration of the strength of this association, we calculated the correlation of the mean AF_VLF_ over the significant voxels as a function of BMI. The AF_VLF_ were significantly positively associated with BMI in areas mostly confined to the anterior cerebellum below the superior cerebellar cistern, and in the brainstem around the area of pre-Bötzinger complex and dorsal and ventral respiratory groups (Fig. 2). In the cerebrum there were significant clusters in thein the left insula and left parietal periventricular white matter. There were small significant clusters at the splenium of the corpus callosum and anterior visual cortex, and in the superior cerebellar cistern.

### Respiratory brain pulsation

**Fig. 3:**
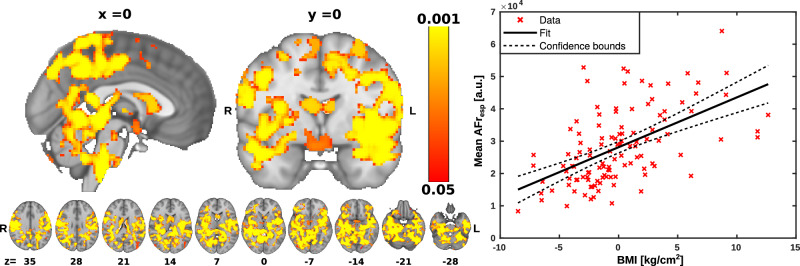
Correlation between body mass index (BMI) and respiration amplitude of fluctuation (AF_resp_) in brain regions. **Left** Statistical map of the voxelwise correlation between AF_resp_ with BMI. AF_resp_ was positively associated with BMI in brain regions indicated on the red-yellow color scale. Significance was assessed using family-wise error rate (FWER) correction at *p* < 0.05. R right, L left, *x* displayed sagittal plane, *y* displayed coronal plane, *z* displayed axial plane. **Right** Correlation plot illustrating the relationship between mean AF_resp_ averaged across significant (*p* < 0.05) brain areas as a function of BMI (R^2^ = 0.3, *p* < 0.001).

To investigate the association between respiratory brain pulsations and BMI, we used the multiple linear regression model to assess voxelwise the association of BMI to AF_resp_. To explore further the strength of this association, we calculated the correlation of the mean AF_resp_ over the significant voxels with BMI. The AF_resp_ were significantly positively associated with BMI in large areas of the brain. Anatomically, the significant areas encompass mostly the posterior sensory and association areas of the brain (Fig. 3). Extending downward, the sensorimotor cortices showed a symmetrical association, and likewise in broad parts of the temporal lobe, including auditory areas and extending to anterior occipital areas close to the V1 visual cortex. There were other bilateral clusters of significant correlation in the lateral ventricles, lateral thalamus areas, splenium of the corpus callosum, and insular cortex. Notably, there was a significant cluster in the hypothalamus just above the stalk of the pituitary, and other clusters in the pons, cerebellum, and brainstem on both sides of the CSF conduits. We also note cluster in the pre-Bötzinger complex of ventral respiratory centers in the caudolateral medulla oblongata.

### Cardiovascular impulse power

**Fig. 4:**
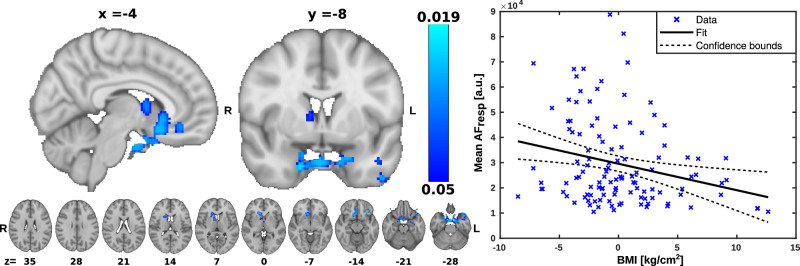
Correlation between body mass index (BMI) and cardiovascular amplitude of fluctuation (AF_card_) in brain regions. **Left** Statistical map of the voxelwise correlation between AF_card_ with BMI, AF_card_ was negatively associated with BMI in brain regions indicated on the blue color scale. Significance was assessed using family-wise error rate (FWER) correction at *p* < 0.05. R right, L left, *x* displayed sagittal plane, *y* displayed coronal plane, *z* displayed axial plane. **Right** Correlation plot illustrating the relationship between mean AF_card_ averaged across significant (*p* < 0.05) brain areas as a function of BMI (R^2^ = 0.046, *p* < 0.05).

To investigate the association between cardiac brain pulsations and BMI, we used the multiple linear regression model to assess voxelwise the association of BMI to AF_card_. To further explore the strength of this association, we calculated the correlation of the mean AF_card_ over the significant voxels and BMI. AF_card_ were significantly negatively associated with BMI in small brain clusters, notably the pituitary gland and stalk, with other clusters in the right caudate nucleus, left medial temporal lobe and and bilateral amygdala (Fig. 4).

### Respiratory modulation of cardiovascular pulsation

**Fig. 5:**
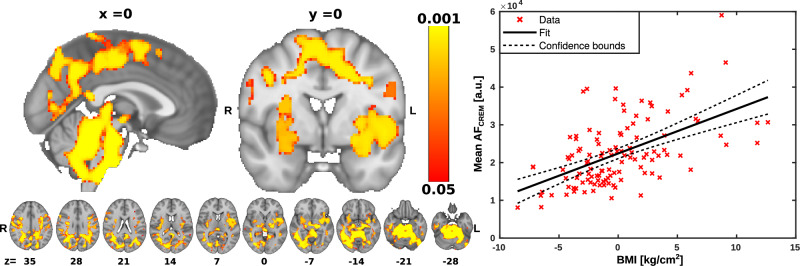
Correlation between body mass index (BMI) and cardiorespiratory envelope modulation (CREM) amplitude of fluctuation (AF_CREM_) in brain regions. **Left** Statistical map of the voxelwise correlation between AF_CREM_ with BMI. AF_CREM_ was positively associated with BMI in brain regions indicated on the red-yellow color scale. Significance was assessed using family-wise error rate (FWER) correction at *p* < 0.05. R right, L left, *x* displayed sagittal plane, *y* displayed coronal plane, *z* displayed axial plane. **Right** Correlation plot illustrating the relationship between mean AF_CREM_ averaged across significant (*p* < 0.05) brain areas as a function of BMI (R^2^ = 0.271, *p* < 0.001).

To investigate the respiratory-induced modulation of cardiovascular pulsation, we used the multiple linear regression model to assess voxelwise the association of BMI to AF_CREM_. To explore further the strength of this association, we calculated the correlation of the mean AF_CREM_ over the significant voxels and BMI. AF_CREM_ had a significant positive correlation with BMI, with a similar voxelwise pattern to that of the AFresp; there were clusters encompassing the upper cerebral sensorimotor cortex and posterior-parietal area, focusing on the grey/white matter junction (Fig. 5). Other significant clusters were around major cerebral arteries, especially in the left insula, and in the whole brainstem and anterior parts of the whole cerebellum on both sides of the IVth ventricle and cerebral aqueducts and again also the pre-Bötzinger complex ja dorsal and ventral respiratory groups. Notably the CSF conduits were not among the significant brain regions included.

## Discussion

Using a fast MREG sequence capable of separating the three known physiological brain pulsations, critically in a manner without aliasing, we aimed to study the relationship between BMI and amplitude of physiological pulsations. Our findings suggest that these pulsations, which are the main drivers of brain solute flow, had strong associations with BMI. The key results were that: (1) AF_VLF_ was positively associated with BMI in brain similar areas as seen in previous studies. (2) AF_resp_ and its cardiovascular modulation AF_CREM_ were positively associated with BMI in widespread posterior parts of the brain. (3) AF_VLF_ and respiratory based pulsations AF_resp_ and AF_CREM_ had a positive association with BMI in brainstem and especially the pre-Bötzinger complex. (4) AF_card_ were negatively associated in the posterior pituitary gland and anterior cerebral artery with BMI.

### Very low frequency vasomotor waves

This study demonstrates a significant association between AF_VLF_ in several specific brain regions and BMI. AF_VLF_ consists of standing oscillations driven by functionally connected neuronal activity, with the most prominent component of AF_VLF_ stemming from propagating vasomotor waves [33]. Recently, these vasomotor waves have also been connected to brain solute transport in the arterial wall and its surroundings [16, 34]. Sleep increases brain solute transport and concomitantly increases the power of vasomotor waves during sleep [17]. Furthermore, the lack of normal sleep/awake homeostasis in narcolepsy was associated with an increased variance of the vasomotor waves [21]. The positive association found in this study between AF_VLF_ and BMI suggests that increased vasomotor wave amplitude may interfere with normal homeostatic brain solute clearance during sleep as a ceiling effect.

In accord with present findings, previous studies using ALFF (a.k.a AF_VLF_ in this study) have found that obese people tended to have increased ALFF in the insula [35, 36], which the authors suggested to indicate increased neural activity in that region. The insular cortex, among many functions, role in the regulation of body weight and energy balance, and has shown hyperactivation in response to food cues and during the consumption of palatable foods in people with obesity [37]. Similarly, activation in the amygdala, which we found to show cardiovascular pulsations correlating with BMI, predicted response to food cues and even to nonfood-related stimuli [38, 39].The splenium of the corpus callosum, which connects the left and right frontal hemispheres of the brain, is reportedly being thinner in people with obesity, possibly indicating a reduction in the integrity of this structure [40]. Thus, we infer in general terms that altered pulsations in association with obesity can evoke structural brain changes.

### The association of respiratory impulse with BMI

We found that the AF_resp_ and AF_CREM_ were both increased as a function of BMI in posterobasal regions of the brain. The significant AF_resp_ correlation encompassed large areas of the brain, especially in the cerebrum, but the modulatory AF_CREM_ correlations with BMI were mainly confined to the brainstem.

Respiratory amplitude fluctuations in the MREG signal arise from opposing venous blood flow and CSF flow pulses during the respiratory cycle, which propagate through all intracranial structures over the respiratory cycle, giving rise to convective CSF and solute pulsations [41]. During inspiration, reduced intrathoracic pressure draws deoxygenated venous blood from the brain veins, and the consequently reduced intracranial blood volume is replaced by inflowing CSF from the spinal canal, thereby keeping constant the intracranial fluid volume constant, in accord with the Monro-Kellie doctrine [13, 42]. During exhalation, the venous withdrawal ceases, and the ballooning cortical veins empty the perivascular (and other) CSF spaces by inflowing capillary blood volume. The outflow of paramagnetic deoxygenated venous blood and inflow of diamagnetic CSF (water) around blood vessels together reduce magnetic spin dephasing due to reduced field distortions, which results in increased T2* signal within brain voxels, especially those in the cerebral cortex [43]. However, respiratory pulsations are present in CSF without venous blood susceptibility changes. These signal alterations may result from T2 flow effects [16]. The predominance of these changes in the posterior fossa near the brain stem may possbily be largely attributed to CSF-related T2 flow effects in these areas.

Numerous studies have linked obesity to various respiratory problems [9]. In physiological terms, excessive weight gain decreases the lung volume, and with increasing mass loading of the chest wall increases, there is a declining respiratory system compliance and higher airway resistance, which together impose a burden of increased muscle workload during breathing [10]. The decrease in overall CSF and cerebrovascular compliance is driven by reduced lung compliance, which in turn arises from increased pulmonary blood volume. Both CSF and paraspinal venous plexus blood volume, as well as their compensatory reserve reductions due to adipose tissue infiltration along the spinal canal, impact cerebrospinal venous blood and hydrodynamics. This, coupled with the need for increased respiratory pressures, results in elevated pressure within both CSF and venous compartments, leading to greater respiratory pulsation changes in the posterior parts of the brain when in a supine position.

Obesity has also an association with neuronal tissue atrophy, especially in white matter [44]. The various physiological restraints associated with obesity affect venous return from the brain while also impeding CSF inflow. Due to the non-compressible nature of fluids, the increased pulsations may directly influence brain hydrodynamics and tissue integrity. Increased intrathoracic pressure can elevate the pressure of respiratory CSF pulsations within the CSF spaces, which are subsequently transmitted to the brain (Fig. 3). Additionally, alterations in pulmonary blood volume can contribute to changes in venous brain pulsations. The increased respiratory intracranial pulsations that we observed, particularly those in the venous system that lacks smooth muscle to absorb pulsations, could in the long term potentially damage brain tissue, thus accounting for tissue atrophy findings cited above. In cases of severe obesity in conjunction with IIHT, CSF dynamics become stagnant, resulting in increased interstitial and perivascular fluid volumes in grey matter, apparently due to increased resistance to cerebral venous drainage [15, 45].

### Brainstem respiratory center effects on breathing and BMI

In this study, the brainstem respiratory centres showed significant associations between BMI and brain pulsations; AF_VLF_, AF_resp_, and AF_CREM_ were all increased in the pre-Bötzinger complex ja dorsal and ventral respiratory centers in relation to high BMI. These pulsations play a role in facilitating interstitial fluid and cerebrospinal fluid (I/CSF) solute transport within brain tissue [46], raising questions about the integrity of solute dynamics in the context of obesity. The satiety hormone leptin potentially links energy and metabolism to breathing by its action at receptors in the brainstem respiratory centers in improving upper airway function during normal sleep [47]. However, patients with obesity have limited leptin permeability across the blood-brain barrier due to transporter receptor resistance and impaired LEPRb signaling, which then attenuates the pro-respiratory effects of the hormone [48]. Another hormone implicated in obesity is orexin, which is involved with both arousal and appetite. Although orexin may have a bidirectional causal relationship with obesity, leptin dysregulation itself can decrease orexin levels in the brain, thereby contributing to the reduced cerebral orexin levels seen in obesity [49]. Orexin neurons of the hypothalamus innervate central autonomic and respiratory regions, including the pre-Botzinger complex as well as phrenic motoneurons, which fall within the brainstem clusters noted in this study [50].

We also observed in this study increased respiratory pulsations in hypothalamic areas and the pituitary gland, especially the neurohypophysis, where we also observed reduced cardiovascular pulsatility, which is generally known to downregulate glymphatic solute transport [46]. Therefore, alterations in leptin and orexin hormonal transport along the I/CSF in obesity may be associated with changes in the brainstem and hypothalamic driving pulsations. The increased pulsatility observed in our study could result directly from the hormonal changes, or may rather serve as a compensatory mechanism for facilitating hormone transport via the paracellular CSF route. Such mechanisms may eventually contribute to establishing a link between narcolepsy (a defect in orexin receptors), sleep apnea, and obesity.

### Cardiovascular pulsations

The AF_card_ was the only endpoint correlating negatively with BMI. Cardiovascular pulsations arise from cardiac induced blood pressure impulses that propagate along the arteries into capillary bed, as well as perpendicularly from the peri-arterial space (Virchow-Robin space) into the brain interstitium. To MREG scanning, the cardiovascular pressure impulse ubiquitously disrupts water proton spin coherence along its path resulting, into a transient drop in T2* signal due to spin-phase and steady-state free precession changes [51].

Obesity adversely affects cerebral arterial function, leading to a decrease in cerebral blood flow, which seems consistent with our present observation of reduced AF_card_ among individuals with higher BMI [52]. Diminished blood flow hampers the delivery of oxygen and glucose to the brain, potentially resulting in the long term in impairment of cognitive functions, including learning and memory [53, 54]. Moreover, an association has been demonstrated between increasing BMI and decreased cardiac output specifically directed to the brain [55].

Obesity is associated with hypertension, and the mechanism has tought to be related to reduced nitric oxide-mediated dilation, along with impaired thalamic insulin signaling, K+ channel function and sympathetic function [56, 57]. Conceptually linking an impaired vasodilation with the inward remodeling process of the arterial wall that reduces the lumen diameter of the cerebral arteries, highlights the potential for chronic hypoperfusion of the brain in obese individuals [54]. Impaired cerebral blood flow also promotes edema formation and the formation of white matter hyperintense T2 signal lesions in MRI. Moreover, reduced arterial wall pulsatility propagates to reduced perivascular solute transport, which can facilitate accumulations of β-amyloid and phosphorylated tau proteins in neurodegenerative pathology [46].

Recent study have shown significant alterations in the propagation of brain cardiovascular pulsations in AD [19]. Furthermore, cerebral blood flow decrease is recognized as an early pathological mechanism in AD [58]. Thus, present observations may serve to link obesity and its association pulsation changes with neurodegenerative diseases.

### Limitations

In this study, we included a diverse range of healthy volunteers extending over a broad range of ages and BMI values; however, we acknowledged that the number of subjects with severe obesity (BMI > 35) was limited. Additionally, we did not assess in our volunteers central obesity, which imparts a higher risk of age-dependent cognitive impairment [59]. As such, incorporating multiple obesity indexes (e.g., waist circumference and full body dual-energy X-ray absorptiometry scans) could provide a more comprehensive exploration of the effects of obesity on physiological brain pulsations. Furthermore, certain factors associated with obesity, such as insulin resistance and plasma leptin levels, were not measured in this study. Additionally, we did not record the duration of obesity, which may have impacted the observed results. Future investigations addressing these aspects are warranted to enhance our understanding of the complex relationship between obesity, physiological brain pulsations, and risk for neurodegenerative changes.

## Conclusion

Our study revealed significant associations between BMI and amplitudes of physiological brain pulsations, especially those related to respiration. In particular, a high BMI was linked to increased respiration-related pulsations in extensive areas of the posterior cerebrum, cerebellum, and brainstem. These findings provides novel insight into pathways through which obesity impacts brain tissue. These increased pulsation amplitudes can induce both direct pressure damage to the tissue integrity and influence brain tissue homeostasis and the functioning of sensitive structures, such as the brain stem respiratory control centers, potentially contributing to the risk for neurodegeneration. Further research is needed to explore the long-term consequences and potential interventions to mitigate the adverse effects of obesity on brain health.

## Data Availability

The data that support the findings of this study are available upon reasonable request from the corresponding author. The data are not publicly available due to privacy and research ethics considerations.
